# Capillary Rise on Legs of a Small Animal and on Artificially Textured Surfaces Mimicking Them

**DOI:** 10.1371/journal.pone.0096813

**Published:** 2014-05-21

**Authors:** Marie Tani, Daisuke Ishii, Shuto Ito, Takahiko Hariyama, Masatsugu Shimomura, Ko Okumura

**Affiliations:** 1 Department of Physics, Faculty of Science, Ochanomizu University, Otsuka, Bunkyo-ku, Tokyo, Japan; 2 Center for Fostering Young and Innovative Researchers, Faculty of Engineering, Nagoya Institute of Technology, Gokiso-cho, Showa-ku, Nagoya, Japan; 3 Department of Life and Materials Engineering, Faculty of Engineering, Nagoya Institute of Technology, Gokiso-cho, Showa-ku, Nagoya, Japan; 4 Department of Biology, Hamamatsu University School of Medicine, Handayama, Higashi-ku, Hamamatsu, Japan; 5 WPI-Advanced Institute for Materials Research (WPI-AIMR), Tohoku University, Katahira, Aoba-ku, Sendai, Japan; 6 CREST, Japan Science and Technology Agency, Hon-cho, Kawaguchi, Japan; Universita’ degli Studi del Salento, Italy

## Abstract

The wharf roach *Ligia exotica* is a small animal that lives by the sea and absorbs water from the sea through its legs by virtue of a remarkable array of small blades of micron scale. We find that the imbibition dynamics on the legs is rather complex on a microscopic scale, but on a macroscopic scale the imbibition length seems to simply scale linearly with elapsed time. This unusual dynamics of imbibition, which usually slows down with time, is advantageous for long-distance water transport and results from repetition of unit dynamics. Inspired by the remarkable features, we study artificially textured surfaces mimicking the structure on the legs of the animal. Unlike the case of the wharf roach, the linear dynamics were not reproduced on the artificial surfaces, which may result from more subtle features on the real legs that are not faithfully reflected on the artificial surfaces. Instead, the nonlinear dynamics revealed that hybrid structures on the artificial surfaces speed up the water transport compared with non-hybrid ones. In addition, the dynamics on the artificial surfaces turn out to be well described by a composite theory developed here, with the theory giving useful guiding principles for designing hybrid textured surfaces for rapid imbibition and elucidating physical advantages of the microscopic design on the legs.

## Introduction

A micropatterned surface that imbibes liquids efficiently would lead to broad technological applications for liquid transport in areas ranging from microfluidics and biomedical mixing devices to fuel transport but has been plagued with a problem that imbibition dynamics generally slows down with time and thus limits applications for long-distance transport. Micropatterned surfaces have been extensively studied [1] for understanding and controlling specific wetting properties such as superhydrophobicity [2], leophobicity [3], and liquid-solid adhesion [4]. Applications include microfluidics devices [5], [6] such as paper-based diagnostic devices [7], liquid-drop transport [8]–[10], controlled and patterned film coating [11]–[13], slippery pre-suffused surfaces [14], [15]. In most of such applications, the key controlling factor is imbibition of patterned surfaces, which has been actively studied for model surfaces on which micro posts are arranged in an array (see, for example [16]). In all previous studies, the dynamics generally slows down with time. Quite frequently, the imbibition length 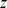 scales with the square root of elapsed time 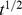 [11], [17], [18], as in the viscous regime of the capillary rise [19], while the dynamics 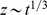 has also been found in different situations [20]–[22]. Similar and other slowing dynamics are also found for imbibition of papers [23]–[25].

Recently, it has been revealed that a small animal, the wharf roach *Ligia exotica*, uses a remarkable micropatterned surface on the legs to absorb water for its survival. However, the imbibition dynamics has yet to be quantified, although remarkable physical features on water transport have been disclosed for living animals, from cats [26], birds [27], to small beetles [28]. Here, we study the imbibition on the legs of the wharf roach to uncover that, although the dynamics is rather complex on a microscopic scale, the imbibition length simply scales with time on a macroscopic scale. Namely, the imbibition dynamics does not slow down, opening a way to achieve long-distance transport by using capillary force (without using a pump). Inspired by the results on real legs, we study artificially textured surfaces mimicking the texture of the animal in a simplified way. The artificial surfaces are comprised of two regions, one decorated with narrow blades and the other with wide blades. As a result we find that the hybrid structure of narrow and wide blades tends to speed up imbibition dynamics compared with the non-hybrid counterparts. We develop a composite theory describing the imbibition of surfaces decorated with blades to find that the theory describes well the imbibition of the non-hybrid surfaces. Furthermore, the initial dynamics on the hybrid surfaces is also explained well by the theory. The theory provides principles useful to design hybrid textured surfaces for swift imbibition and clarifies physical benefits of the remarkable design on a microscopic scale found on the legs.

## Microscale Texture on the Legs of a Wharf Roach

**Figure 1:**
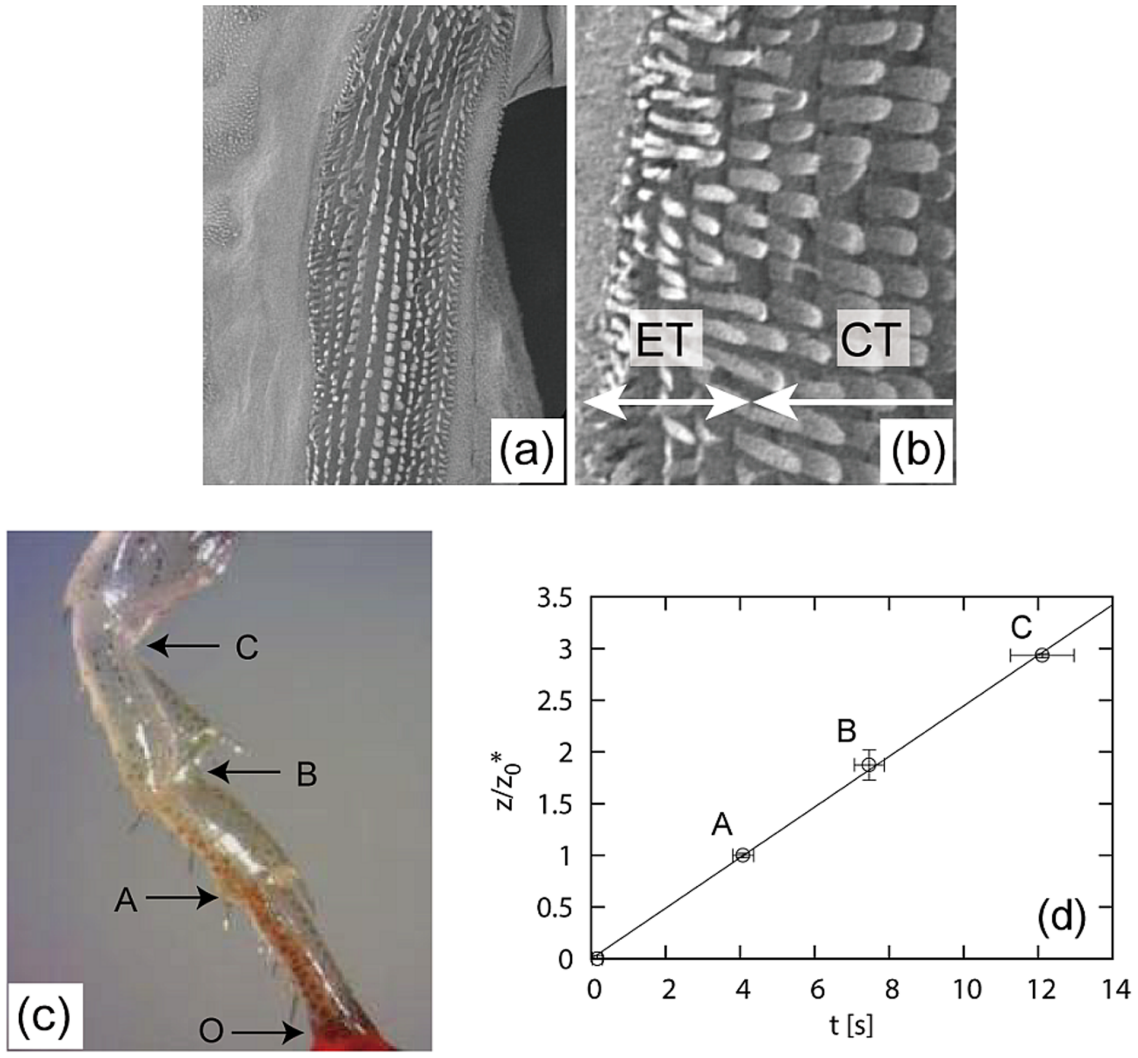
(a)–(b) SEM images of a leg of the wharf roach *Ligia exotica*. A path for water transport on the leg seen in (a) is covered with small blades. In a simplified view, wide blades cover the central-texture (CT) region and narrow blades the edge-texture (ET) region, as seen in the image in (b) showing the boundary between the central and edge regions with a higher magnification. The height of the blades are about 50 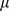
m. (c) A leg of the wharf roach used for the imbibition experiment. (d) Height 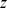 of the rising water front, renormalized by the length 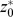 of the lowest podite in (c), as a function of the elapsed time 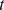. The labels in the plot correspond to those in (c). Label 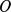 in (c) corresponds to the origin of the plot.

The wharf roach *Ligia exotica* (Crustacea, Isopoda) is a small animal living by the sea which runs away fast often in a group when we walk on breakwaters. To survive it absorbs water from the sea through its legs [29]. On the outer surface of the leg there is a path for water transport covered with a remarkable array of small blades of micron scales as in Fig. 1a–b [30]. In a simplified view, two types of blades are arranged parallel to the direction of water transport: wide blades are placed near the center of the path and narrow blades are ordered near the edges surrounding the central region. The path is disturbed by joints where the path is covered with narrow blades.

## Capillary Rise on the Path on the Legs

When a tip of the leg is touched with a horizontal surface of a water bath, water rises along the path against gravity, similarly as on artificial surfaces with an array of micro pillars. A snapshot is shown in Fig. 1c.

The rising motion of the propagating front is rather complex on a microscopic scale but can be quantified on a macroscopic scale. In the complex dynamics, the front is not a simple horizontal line and sometimes branches off even with one of the branches starting to flow backward. This may be caused by irregularity of the texture, while the slits between the blades seemingly give robustness for water transport, allowing lateral transport. Interestingly, the front seems to start proceeding to the next podite (a part between two adjacent joints) only after the first podite is almost filled. Even if the filling dynamics within a podite is complex, the moment when the bulk water front arrives at each joint (to proceed to the next podite) is rather well defined. These moments and the positions of corresponding joint are quantified in Fig. 1d.

As shown in the figure, the imbibition height 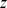 thus macroscopically obtained scales with the time 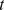, which is quite unusual in terms of imbibition dynamics, whose speed usually slows down with time. The non-slowing-down linear dynamics 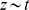 on a macroscopic scale seems to result from repetition of unit dynamics over each podite: the dynamics consisting of repeating units might be advantageous for long-distance transport.

## Artificially Textured Surfaces Mimicking the Animal’s Leg

**Figure 2:**
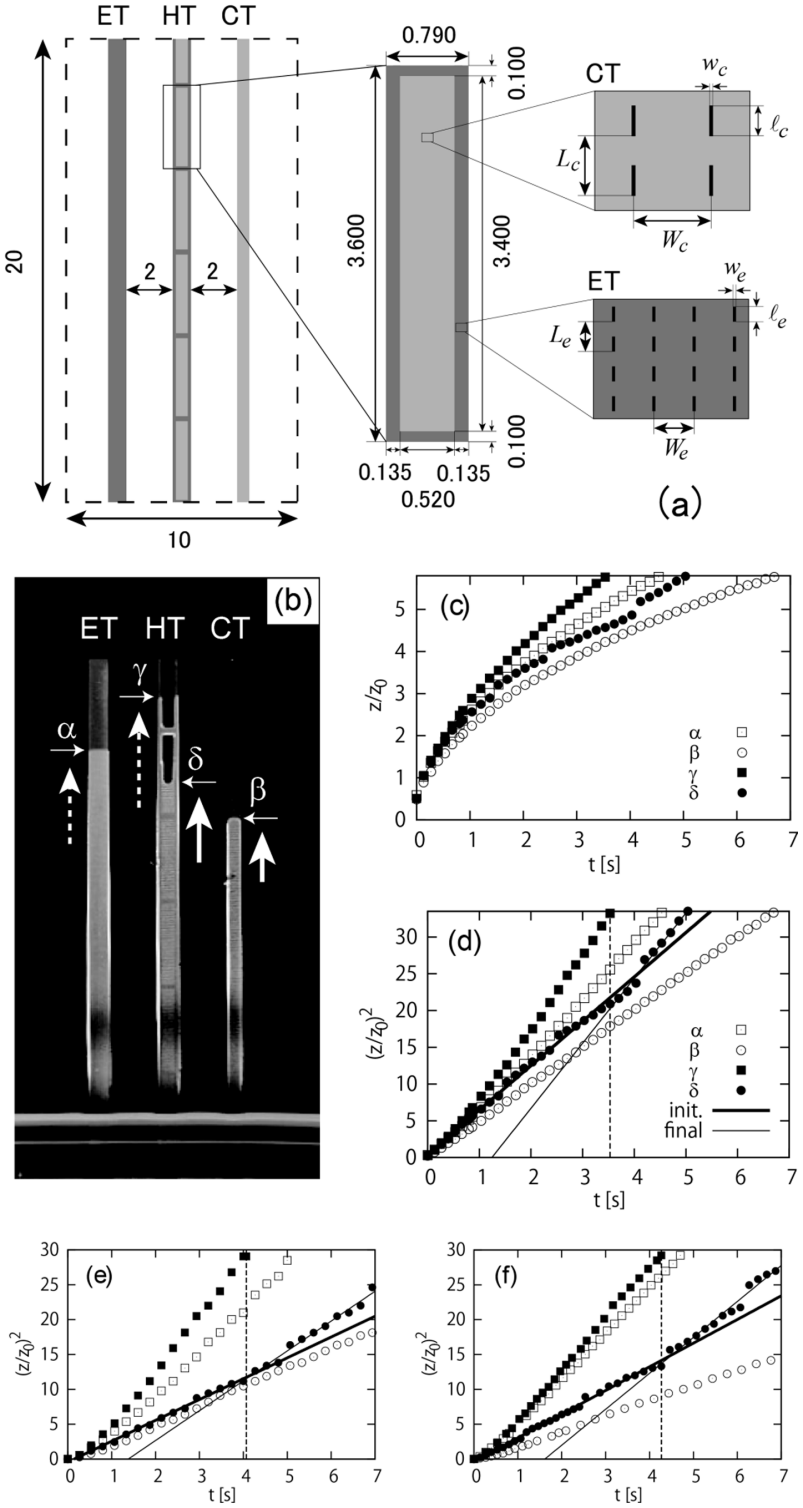
(a) Representative textured pattern mimicking the surface of legs of the wharf roach *Ligia exotica*. The right and left paths mimic the central and edge regions, while the middle path is a hybrid of central and edge textures. The length of “podite” is designed to be 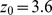 mm (or 3.4 mm), which is comparable to a typical size of podite in real legs. The sizes in the illustrations are expressed in the unit mm. (b) A snapshot of capillary rise on the reference surface. The imbibition fronts are shown by the horizontal arrows. The vertical arrows indicate velocities with reflecting relative magnitudes. (c) Height of the rise 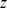 renormalized by 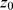 vs elapsed time 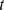 obtained from the reference surface. The labels, 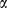-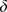, correspond to the labels in (b). (d) 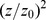 vs 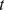, obtained from the reference surface. The solid and thin lines with Labels “init.” and “final” are those fitting the data in the initial and final regions of the front in the central region of the HT path labeled 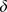 in (b). (e) and (f): the same plots as the plot in (d) but for substrates different from the reference.

Motivated by the unusual features of the capillary rise on the legs discussed above, textured surfaces mimicking the texture on the legs are fabricated as illustrated in Fig. 2a on a silicon wafer by photolithography, and the surfaces are irradiated by deep ultraviolet light to achieve completely wetting state for water. The three paths are called edge-texture (ET, left), hybrid-texture (HT, middle) and central-texture (CT, right) paths. The textures on the ET and CT paths mimic those in the edge and central regions of the path of the legs. The HT path consists of the edge and central textures where five and a half central-texture regions are separated by “joints” which are covered with the edge texture, as shown in Fig. 2a left. The central and edge textures are specified, as in Fig. 2a right, by the set of lengths 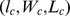 and 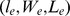 of micron scales, with the height 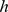 and thickness 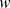 (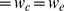) of the blades fixed to 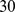 and 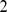 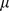
m, respectively.

Faithful mimicking of real legs is technically difficult. We fabricated a three-path pattern whose length scales are given by the set 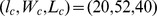 and 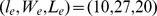 in the unit 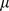
m. This set corresponds to typical values for real legs. By taking this pattern as a reference pattern, we fabricated patterns with different parameters. However, the real blades are too high and thin for practical fabrication; we set 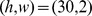 in 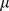
m for all the samples as above, although typical height and thickness of blades are 50 and less than one 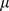
m, respectively. Furthermore, the blades may be bendable with capillary force, and some are placed on the base with some tilt angles. In addition, the real structure is not so regular as in the imitations, while the complex dynamics on the scale of blades seems to be strongly dependent on the irregularity.

## Imbibition of the Reference Surface

A snapshot of the imbibition of the reference substrate is shown in Fig. 2b. As seen here, on artificial surfaces, the imbibition front is practically a horizontal line, because of the regularity of the pattern. The dynamics obtained from the reference pattern is quantified in Fig. 2c. We track the imbibition fronts on the ET (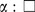) and CT (*β*:○) paths, and in the edge (*γ* :▪) and central regions (*δ* : •) of the HT path, as indicated in Fig. 2b.

As seen from Fig. 2c, imbibition on the HT path of the reference surface shows some remarkable features: imbibitions in the edge and central regions on the HT path are both made faster than the original non-hybrid counterparts. (1) Imbibition on the ET path (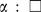) is faster than that on the CT path (*β*:○) because a typical size of blades is smaller on the ET path: capillary drive is stronger for textures with smaller blades (stronger viscous friction is less dominant as shown in the theory below). (2) The propagating front in the edge region of the HT path (*γ* :▪) is even faster than the front on the ET path (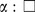) as indicated by the two dashed vertical arrows drawn on Fig. 2b, although the values of the set of parameters 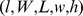 are the same for both. This is because on the HT path the moving water front in the central region plays a role of the liquid bath for imbibition in the edge region. (3) The front in the central region of the HT path (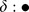) is faster than that on the CT path (*β*:○) as indicated by the two solid vertical arrows drawn on Fig. 2b because the preceding water in the edge region gives an extra pull for the front in the central region.

In Fig. 2d, the plot of 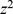 as a function of 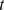 suggests that the 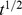 dynamics is valid at least for the ET and CT paths (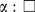 and *β*:○). Though rather technical, the origin of 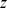 in Fig. 2c–f is shifted by a small amount, corresponding to an initial dip length to cause the imbibition, in order to clarify the dynamics in the unit 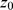. This shift is much smaller than the overall imbibition length and does not affect practically the linear relation between 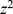 and 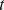.

Also seen in Fig. 2d, the imbibition in the central region of the HT path (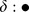) is divided in the initial and final dynamic regimes, as indicated by the solid and thin lines, with the vertical dashed line separating the two regimes. The initial dynamics proceeds roughly as 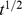 though intermittent at the “joints,” and is slightly faster than the dynamics on the CT path (*β*:○). The final regime starts when the front on the edge region of the HT path (*γ* :▪) reaches the top of the pattern, as indicated by the vertical dashed line.

The imbibition dynamics on the artificial substrates are rather different from those on the legs: the imbibition speed slows down but an average speed is faster on the artificial substrates. Although the dynamics slows down on the artificial surfaces, the time required for the water front to travel over the first three “podites” in the reference surface (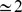 sec) is several times as short as the corresponding time on real legs (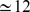 sec). In other words, the structure on real legs may not be optimized in terms of the speed of imbibition.

Several dynamic features observed in Fig. 2b–d on the reference substrate qualitatively remain the same on substrates different from the reference, as seen in Fig. 2e and f that are obtained from substrates specified by a set of central parameters 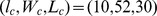 and another set 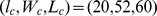, respectively, while the set of edge parameters 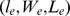 are both the same as on the reference substrate, i.e., 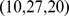. In particular, the imbibition dynamics in the central region of the HT path is always divided in the two regimes, (1) the initial intermittent dynamics slightly faster than that on the CT path, and (2) the final faster dynamics starting when the edge front reaches the top. This suggests (1) the initial dynamics on the HT path may be described by the same theory as a theory developed for the simpler CT path, which will be confirmed below, and (2) we could speed up the imbibition dynamics if we could stop the water front at each joint as on real legs because such a temporal stop may cause a speedup, as seen at the dynamic transition from the initial to final regimes, as demonstrated in Fig. 2d–f.

## Composite Model of Imbibition Dynamics

To quantify our observation, we develop a theory describing the imbibition on the CT path, which should also be applicable to the imbibition on the ET path because geometries on the both paths are the same. The construction of the theory is performed by extending the scaling arguments given in [17]. The thickness of the imbibed film is expected to be comparable to the height of blades 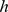 when 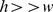 as in the present case, as discussed in [31] and indirectly proved in [17].

The capillary drive per unit width of the film is then estimated as 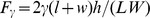, where 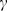 is the surface tension of the liquid (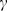 in Fig. 2 should not be confused with this); this is obtained from the change in the surface energy per unit width of the liquid film due to the displacement 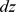 of the film front 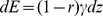 where the roughness is defined as 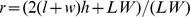. Here, the set 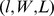 corresponds to 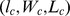 and 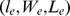 on the CT and ET paths, respectively.

We consider two types of viscous friction acting on the film and construct the total friction as a composite of the two. The texture on the CT and ET paths can be regarded as a stripe of two regions, one with blades and the other without blades. In the region without blades, the friction on the film comes from the bottom surface of the pattern: 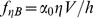 per unit area of the film, with 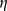 the viscosity of the liquid and 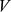 the velocity of the front of the liquid column. In the region with blades, in addition to the bottom friction, the friction with the side walls of the blades, 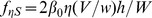, comes into play, where the factor 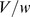 is due to the velocity gradient and the factor 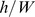 represents the normalized area for which this friction force takes place. In the above, 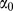 and 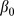 are numerical coefficients roughly of the order of unity. However, when 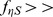 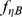, i.e., 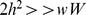, only the side wall friction is important in the region with blades. In such a case, the total friction 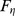 is given as a composite of the two types of friction on each region, i.e., 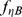 in the region without blades and 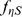 in the region with blades: 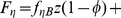 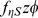, where the fraction of the region with blades is given by 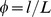.

By balancing 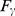 with 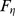 we obtain
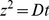
(1)where



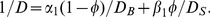
(2)Here, 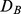 and 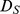 are given as
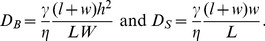
(3)and 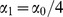 and 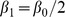 are numerical coefficients roughly of the order of unity.

A number of practical guiding principles can be obtained from these results and the theory elucidates physical benefits of the microscopic design on the legs. In order to speed up imbibition, we need to increase 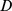 with satisfying the assumptions of the theory:
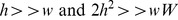
(4)


One efficient way is to make 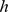 larger, as realized on the real legs. For efficient water transport we may have to consider other factors. For example, to keep enough flux the condition 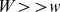 should be important, also as realized on the real legs.

For later convenience, we note here two relations derived from above equations:
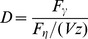
(5)

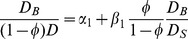
(6)


## Comparison of the Data with the Theory

**Figure 3:**
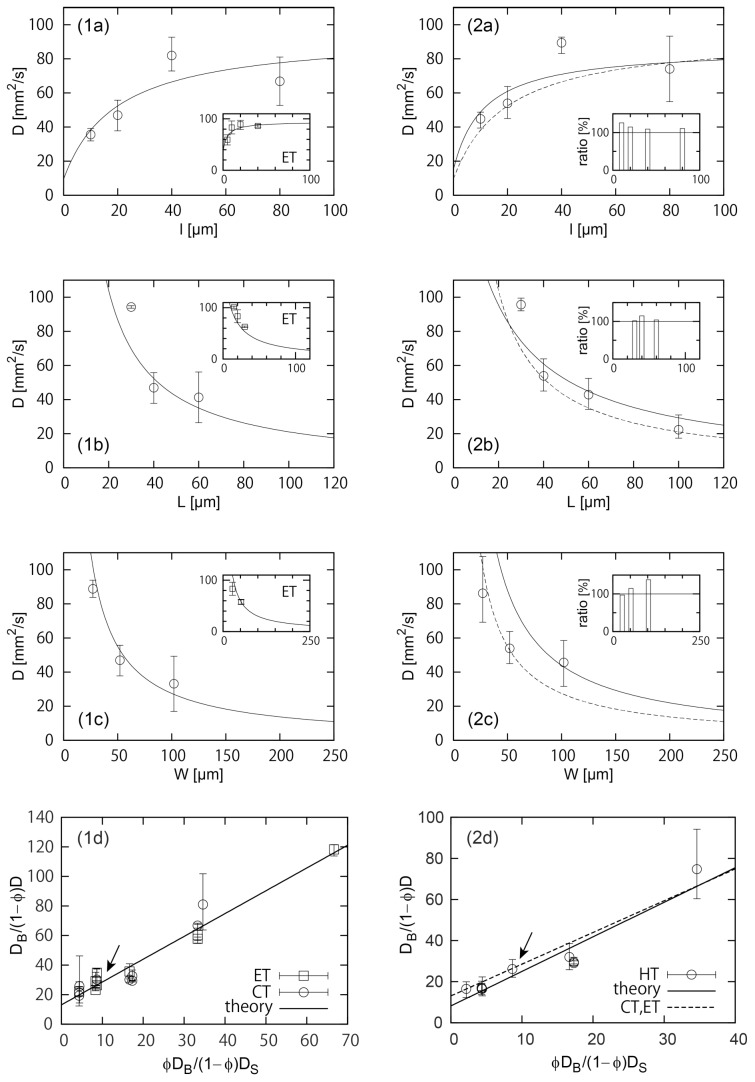
(1a)–(1d) Texture-parameter dependences of the imbibition dynamics on the CT paths, with the insets giving those on the ET paths. (1a) The coefficient 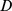, a measure of the rising speed, vs the length 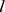 at 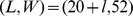 for CT and 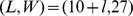 for ET. (1b) 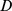 vs 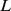 at 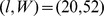 for CT and 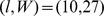 for ET. (1c) 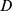 vs 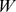 at 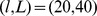 for CT and 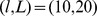 for ET. (1d) Comparison of the data with the theory. All the data collected from (1a) - (1c), together with other three data, are well on a straight line as predicted in Eq. (6). The straight line shown in the plot is obtained by numerical fitting. The curved lines in (1a)–(1c) and those in the inset are based on the theory in Eq. (6) with 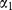 and 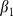 determined by the straight line in (1d). (2a)–(2d) Texture-parameter dependences of the “initial” imbibition dynamics in the central region of the HT paths with the edge region specified by the set 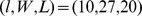. (2a) 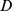 vs 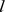 at 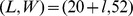. (2b) 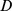 vs 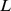 at 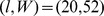. (2c) 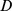 vs 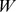 at 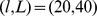. (2d) Comparison of the data with the theory. All the data collected from (2a)–(2c) are well on the straight solid line, obtained by numerical fitting. The curved solid lines in (2a)–(2c) are based on the theory in Eq. (6) with 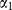 and 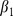 determined by the straight solid line in (2d). The insets to (2a)–(2c) quantify speed-up by the edge effect, showing the ratios of average values of 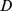 in the main plots of (2a)–(2c) to those in (1a)–(1c), demonstrating the dynamics in the central region on the HT paths are clearly faster than those on the corresponding CT paths, except for two exceptional cases. The dashed lines in (2a)–(2d) are the solid lines in (1a)–(1d), respectively, confirming the edge effect in different ways.

To test the above theory, we plot the coefficient 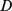 obtained from the 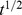 dynamics observed on the ET and CT paths for various 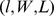 in Fig. 3 1a–1c. Note that the assumptions of the theory given in Eq. (4) are well satisfied in the data. Although rather technical, note also that, in obtaining the coefficient 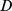, the small artificial shift introduced for Fig. 2c–f is not invoked: 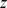 is the imbibition length in the strict sense.

The systematic trends in Fig. 3 1a–1c are consistent with the above theory. The dependences of 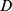 are almost governed simply by change in the driving force 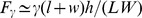, as mentioned above, although, in principle, the dependences of 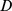 on 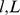, and 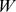 are determined by complex balance of changes in the driving force 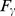 and friction force 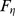: the increase in the driving force tends to increase the speed, thus increase 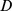, while that in the friction force tends to decrease 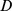. For example, with increasing 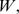 the driving force 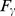 decreases, which results in the decrease in 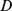 shown in Fig. 3 1c, although the friction force 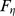 decreases. This dominance of the driving force is consistent with the structure of Eq. (5): different from 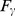 in the numerator, 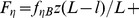 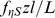 in the denominator is composed of two terms 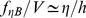 and 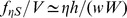 which are differently dependent on the lengths.

For completeness, we here discuss how we estimated the error bars in this study. We used substrates, each of which can be specified by six parameters 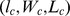 and 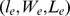. We fabricated non-reference substrates in such a way that only one parameter is changed in the six parameters of the reference substrate, or all the six parameters are changed at once to 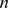 (or 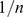) times as large as those of the reference (with 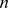 an integer), etc. As a result, if we perform imbibition experiment *once per substrate* (while each substrate has three paths, ET, HT, and CT on it), numbers of data sets that are obtained for different parameter sets (of ET, HT, or CT) become different in a complex way. We thus estimate error bars in the following way: (1) When more than 10 data sets are available for a certain blade parameter (of ET, HT, or CT), we estimate the error bars as the standard deviation: the error bars become symmetric with the mean. (2) When the number of data sets is less than 10, the error bars are estimated from the maximum, minimum, and the mean: the error bars could become asymmetric. The principle origin of the error bars is not in the difficulty of determining the coefficient 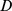 from the 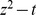 plot, but in the difficulty of attaining a homogeneously complete wetting state for water because the effect of the irradiation of deep ultraviolet light is not robust (the light treatment and the imbibition experiment were performed in different labs).

A more quantitative confirmation of the validity of the theory is shown in Fig. 3 1d, where Eq. (6) is tested. As predicted, the data are well on the straight line with 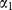 and 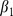 being 13.1 and 1.54 (roughly of the order of unity as expected). With these values of 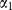 and 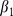, the composite theory well describes the systematic trends in Fig. 3 1a–1c, as indicated by the theoretical curves.

In the above, we expect that the initial “intermittent” 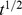 regime of the imbibition dynamics observed in the central region of the HT path is explained by a theory developed for the dynamics on the CT path, if we are allowed to neglect the effects of the edges (and joints). This is confirmed in Fig. 3 2a–2d. The systematic trends for change in each length in Fig. 3 2a–2c are quite similar to those in Fig. 3 1a–1c. Figure 3 2d confirms the validity of the theory more quantitatively, showing a linear relation between the quantities in the horizontal and vertical axes (solid line with 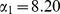 and 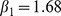), as predicted in Eq. (6). Note that the present composite theory does not include the effect of the joint region. The theory still describes well the dynamics in the central region of the HT path because the fraction of the joint regions are quite low.

Although the dynamics in the central region of the HT paths can be explained well by the theory, the dynamics are faster than the corresponding dynamics on the CT paths, due to the edge effect (including the effect of joints on which the edge pattern is present). This speed-up effect is clearly visible in the insets in Fig. 3 2a–2c, except for two exceptional cases, in which 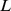 or 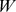 is small: it seems that the edge effect tends to play an efficient role when the length scales in the edge pattern are small enough compared with those in the central pattern, as on real legs. The comparison of the solid and dashed curves also show the speed-up effect (the second-left data points in (1a)–(1c) and (2a)–(2c), and the circles with arrows in (1d) and (2d) correspond to the reference substrate). This edge effect is further understood in a systematic way from Fig. 3 2d: the edge effect measured through 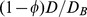 increases as 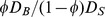 decreases.

## Conclusion

We found that the imbibition on the legs of the wharf roach *Ligia exotica* proceeds with linearly in time on the scale of podite. The unusual dynamics advantageous for long-distance water transport seems to result from repetition of unit dynamics. We were biologically inspired to study artificially textured surfaces mimicking the structure on the legs of the animal simply as a hybrid structure of two types of texture, one with narrow blades and the other with wide blades.

We found that the imbibition of the hybrid textured surfaces is not linear, which is different from the case of real wharf roaches. This disagreement might result from the combination of the following features on the real ones: (1) The real structure is rather inhomogeneous, possibly with gradients of the texture and wettability. (2) The width of the path is also not homogeneous over the podites. (3) The blades are higher and thinner, and, probably, bendable. In addition, blades are sometimes tilted on the surface. These features may lead to the complex imbibition on the microscopic scale in which the front seems to start proceeding to the next podite only after the first podite is almost filled; This “ waiting” behavior might lead to the liner dynamics. We have yet to clarify why the liner dynamics results in the case of natural wharf roach in the near future.

For the nonlinear dynamics on the artificial surfaces, the dynamics in the central region of the hybrid surfaces (HT) is faster than that on the non-hybrid surfaces (CT and ET). In addition, we developed a composite theory for the imbibition of the surfaces textured with blades. The theory explains well the initial dynamics on the hybrid surfaces, in addition to the dynamics on the non-hybrid surfaces. As a result, the theory gives guiding design principles for hybrid textured surfaces which realize quick imbibition. In addition, advantages of the design principles employed in the real legs are supported by the theory.

Faithful mimicking of the real structure on the legs will be a challenging future problem towards artificial realization of the linear dynamics. The texture on real legs may not be optimized in terms of speed because the average speed of imbibition is faster on artificial surfaces, but provides ideas for liquid transport by surfaces textured with hybrid patterns: (1) The edge region may contribute to speeding up the imbibition dynamics because the faster imbibition at the edges assists the slower imbibition in the central region. (2) Temporal stops of imbibition at the joints may speed up the dynamics, as demonstrated by the speedup associated with the dynamic transition on the HT path. (3) By virtue of the faster imbibition at the edges, the joints tend to be filled before the arrival of the front in the central region, which also contributes the speed-up. (4) The slits between the blades may provide robustness for water transport, allowing horizontal transport in addition to vertical transport for capillary rise. These ideas will be useful for developing efficient pump-less devices for liquid transport in various fields in the near future.
